# Genomic Evolution and Patterns of Horizontal Gene Transfer in Coccomorpha Species

**DOI:** 10.1002/ece3.72158

**Published:** 2025-10-09

**Authors:** Yi‐Xin Huang, Ji‐Mu Lv, Cheng‐Yang Ding, Xin‐Yi Zheng, Xing‐Jie Liu, Xiao‐Nan Chen, Shun‐Hai Yu, Yin‐Feng Meng, Hao‐Yuan Hu, Xu Wang

**Affiliations:** ^1^ Collaborative Innovation Center of Recovery and Reconstruction of Degraded Ecosystem in Wanjiang Basin Co‐Founded by Anhui Province and Ministry of Education, School of Ecology and Environment Anhui Normal University Wuhu China; ^2^ Institute of Entomology Guizhou University/Guizhou Provincial Key Laboratory for Agricultural Pest Management of the Mountainous Region Guiyang China; ^3^ The Key Laboratory for Silviculture and Conservation of Ministry of Education Beijing Forestry University Beijing China; ^4^ Qianjiangyuan National Park Administration Quzhou China; ^5^ College of Biology Pharmacy and Food Engineering Shangluo University Shangluo China; ^6^ Anhui Provincial Key Laboratory of the Conservation and Exploitation of Biological Resources, College of Life Sciences Anhui Normal University Wuhu China

**Keywords:** Coccomorpha, comparative genomic analysis, detoxification‐related genes, HGT, phylogenetic analysis

## Abstract

As a significant group of agricultural and forestry pests, Coccomorpha warrants in‐depth investigation into their environmental adaptation mechanisms. This study conducted a comparative genomic analysis using five published chromosome‐level genomes of Coccomorpha species. Phylogenetic analysis revealed that the divergence times of these five species ranged from 333.18 to 84.22 million years ago (mya), with each having undergone two whole‐genome duplication (WGD) events. The significantly expanded gene families in these species were predominantly enriched in antioxidant‐related processes such as oxoacid metabolic process, organic acid metabolic process, and carboxylic acid metabolic process. Furthermore, 260 horizontal gene transfer (HGT) acquired genes were identified across these species, primarily originating from bacteria and archaea. These HGT‐acquired genes were mainly involved in nutrient metabolism, suggesting their role in enhancing nutritional acquisition and metabolic flexibility. Through systematic identification of detoxification‐related genes, ATP‐binding cassette (ABC), carboxylesterases (COE), cytochrome P450, and UDP‐glucuronosyltransferases (UGT) were identified as the major detoxification gene families in Coccomorpha, with significant variations in gene number and composition among different species. This study provides comprehensive insights into the genomic adaptations of Coccomorpha species, highlighting the roles of gene family dynamics, HGT, and detoxification mechanisms in their evolutionary success. These findings offer resources for understanding the molecular basis of Coccomorpha adaptation and provide references for developing targeted pest management strategies.

## Graphical abstract

This study reveals the genomic adaptations of Coccoidea pests through comparative analysis of five chromosome‐level genomes. Key findings include whole‐genome duplication events, expansion of antioxidant‐related gene families, horizontal gene transfer for nutrient metabolism, and species‐specific detoxification mechanisms linked to pesticide resistance. These insights advance understanding of Coccoidea's evolutionary success and provide a foundation for targeted pest control strategies.

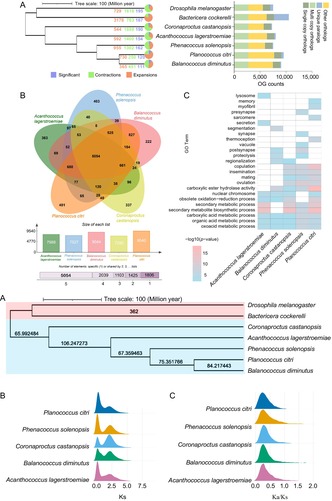

## 1 Introduction

Coccomorpha, commonly known as scale insects, are a superfamily within the order Hemiptera and suborder Sternorrhyncha (Kondo et al. 2009). As highly specialized plant parasites, they comprise over 8000 described species and are widely distributed across tropical, subtropical, and temperate regions (Gullan 2007). Most scale insects exhibit extreme sexual dimorphism and distinct life history traits, with sessile adult females forming dense colonies on their host plants, while the short‐lived winged males are relatively limited in dispersal due to poor flight ability and brief adult lifespan. In contrast, the crawler‐stage nymphs are more mobile and play a major role in population spread, with some species capable of passive dispersal via wind or animal‐mediated transport (Hodgson and Henderson 2004).

Scale insects feed on plant sap using piercing‐sucking mouthparts, directly damaging plant tissues and affecting host vigor (Howard et al. 2001). Additionally, their excretion of sugar‐rich honeydew creates favorable conditions for the growth of sooty mold fungi, which block photosynthesis and further weaken host plants (Kapranas 2012). Moreover, some species act as vectors of plant pathogens, amplifying their overall impact (Ross et al. 2010). Coccomorpha infestations are widespread in agriculture, horticulture, and forestry, causing considerable economic losses. For example, *Quadraspidiotus perniciosus* infests more than 230 plant species, including pear, apple, peach, and grape (Verma and Dinabandhoo 2005); *Phenacoccus solenopsis*, first reported in China in 2009, now affects at least 166 host species, notably in the Malvaceae, Solanaceae, and Euphorbiaceae families, threatening crops such as cotton and vegetables (Chen et al. 2023); *Ceroplastes stellifer* damages over 100 plant species from 30 families, including mango and citrus, and is now reported in nearly 60 countries across Asia, the Americas, and Oceania (Velez‐Gavilan, 2022).

Throughout their long evolutionary history, Coccomorpha have developed unique adaptive mechanisms, which are reflected not only in their physiological structures but also in their interactions with the environment. For instance, most Coccomorpha typically adopt a sedentary lifestyle, firmly attaching themselves to plant surfaces and secreting waxy protective coverings to shield against environmental stressors and natural enemies (Dhooria 2008). Additionally, Coccomorpha exhibit a high degree of host plant specificity. This specificity may result from non‐adaptive factors during historical evolutionary processes rather than simple adaptive selection (Peterson et al. 2020). Such characteristics enable Coccomorpha to thrive and reproduce successfully within specific ecological niches, while simultaneously limiting their ability to expand into different environments. In‐depth research into these adaptive mechanisms of Coccomorpha can provide new insights and approaches for the integrated management of agricultural and forestry pests.

At the molecular level, HGT is a significant phenomenon in biological evolution and plays a crucial role in the adaptive evolution of insects. These HGT‐acquired genes are likely involved in critical biological processes such as metabolic regulation, immune defense, and environmental adaptation in the insect. Research indicates that HGT‐acquired genes may participate in comprehensive physiological metabolism, potentially contributing to functional innovation and adaptation within ancient lineages of insect hosts (Li et al. 2011). For example, *Bemisia tabaci* has acquired functional carbohydrate‐active enzymes from plants through HGT (Colinet et al. 2025), and HGT‐acquired genes have even been shown to enhance courtship behavior in Lepidoptera insects (Li et al. 2022).

Insects have developed sophisticated detoxification mechanisms to cope with plant secondary metabolites and insecticide stress. These mechanisms primarily rely on a series of detoxification‐related gene families, including cytochrome P450, glutathione S‐transferases (GSTs), COEs, and so on. Among these, P450 enzymes are renowned for their genetic diversity and broad substrate specificity, playing a critical role not only in the metabolism of endogenous compounds but also in the detoxification of exogenous substances. This contributes to the development of insect resistance to synthetic insecticides and enhances their adaptation to plant chemical defenses (Feyereisen 2012). Meanwhile, insecticide‐induced GSTs can act as antioxidants to protect insects from oxidative stress (Vontas et al. 2001), while COEs often increase insecticide resistance through mechanisms such as gene coding sequence mutations, constitutive overexpression, induced expression, or a combination of these strategies (Li et al. 2007). In‐depth research into these detoxification mechanisms not only advances our understanding of insect adaptive evolution but also provides a theoretical foundation for developing novel pest control strategies.

In recent years, the rapid advancement of genomic technologies has significantly enriched the genomic resources of Coccomorpha. The genomes of multiple scale insect species have been published, including *Acanthococcus lagerstroemiae*, *Coronaproctus castanopsis* (Huang et al. 2024), *Planococcus citri* (Ross et al. 2024), *Balanococcus diminutus* (Ross and Watson 2024), and *P. solenopsis* (Li et al. 2020). The release of these genomic datasets provides a valuable foundation for in‐depth research into the biological characteristics, evolutionary mechanisms, and environmental interactions of scale insects. However, studies on the adaptive evolutionary mechanisms of Coccomorpha remain relatively limited, which hinders a comprehensive understanding of their biological traits and the development of effective control strategies. This study aims to elucidate how gene family dynamics, horizontally transferred genes, and the evolution of detoxification‐related genes contribute to the environmental adaptation of Coccomorpha species. Through comparative genomic analyses of five representative species, this study reconstructed their evolutionary relationships and divergence times, investigated patterns of gene family expansion and contraction, identified genes acquired via HGT, and characterized the functional evolution of detoxification‐related gene families. Together, these findings provide novel insights into the adaptive evolution of scale insects, particularly in relation to pesticide stress and ecological specialization.

## 2 Materials and Methods

### 2.1 Data Sources

Genomic data were obtained from the NCBI database (https://www.ncbi.nlm.nih.gov/), encompassing five Coccomorpha species: *Acanthococcus lagerstroemiae* (GCA_031841125.1), *Coronaproctus castanopsis* (GCA_032883995.1), *Planococcus citri* (GCA_950023065.1), *Balanococcus diminutus* (GCA_959613365.1), and *Phenacoccus solenopsis* (GCA_009761765.1). The genomes of two outgroup species (*Bactericera cockerelli* and *Drosophila melanogaster* ), used for phylogenetic analysis, were retrieved from InsectDB (https://v2.insect‐genome.com/).

### 2.2 Phylogenetic Analysis of Single‐Copy Orthologs in Coccomorpha

To improve the accuracy and stability of the phylogenetic tree, *B. cockerelli* and *D. melanogaster* were selected as outgroup species, as they are phylogenetically close to Coccomorpha but fall outside the Coccomorpha. OrthoFinder v2.5.5 (Emms and Kelly 2019) was employed to identify single‐copy orthologous genes across five Coccomorpha species and two outgroups. Multiple sequence alignment of the single‐copy orthologs was performed using MAFFT (v7.475) (Katoh and Standley 2013), followed by trimming of low‐quality alignment regions with trimAl (v1.4.rev22) (Capella‐Gutiérrez et al. 2009) (paramenters: “‐automated1”). The trimmed sequences were subjected to Maximum Likelihood (ML) phylogenetic analysis using RAxML (v8.2.12) (Stamatakis 2014) under the PROTGAMMAJTT model, with 1000 bootstrap replicates to ensure the reliability of the tree.

To estimate divergence times among species, r8s (v1.81) (Sanderson 2003) was utilized. The species tree generated by OrthoFinder was first converted into NEXUS format and input into r8s. The divergence time between *Bactericera cockerelli* and *D. melanogaster* , obtained from the Timetree web service (http://www.timetree.org/), was set at 362 million years as a calibration point. r8s calculated the divergence times of each node using the Penalized Likelihood method and generated an ultrametric tree. The final phylogenetic tree and divergence time results were visualized and annotated using FigTree (v1.4.4) (http://tree.bio.ed.ac.uk/software/figtree/).

### 2.3 Identification of Orthologs and Paralogs and Calculation of the Synonymous Substitution Rate (Ks) and Nonsynonymous Substitution Rate (Ka)

To identify orthologous and paralogous genes, self‐alignments and cross‐species protein alignments were first performed for each species using BLASTP (Li et al. 2015). A BLAST database was constructed for the protein sequences of each species using makeblastdb, followed by self‐alignments and cross‐species alignments via BLASTP (parameters: “‐outfmt 6 ‐evalue 1e‐5 ‐num_descriptions 10”). To ensure the validity of the analysis, self‐alignment results were filtered to remove self‐hit entries, retaining only alignments between different genes. The best‐matched gene pairs from self‐alignments were identified as paralogs, while the best‐matched gene pairs from cross‐species alignments were identified as orthologs. The Ks and Ka were calculated using KaKs_Calculator (Zhang 2022). Finally, density distribution plots of Ks values were generated using the ggplot2 (Wickham and Chang 2016) and ggridges (Wilke 2024) packages in R.

To further investigate the selective pressure acting on protein‐coding genes, codon‐based selection analysis was performed using HyPhy (Pond et al. 2005). Specifically, single‐copy orthologous gene alignments were used as input for site‐level selection analysis with the SLAC (Single Likelihood Ancestor Counting) model in HyPhy, which estimates the ratio of nonsynonymous to synonymous substitution rates (dN/dS) at each codon site. Sites under purifying or positive selection were identified based on significance thresholds (*p* < 0.1), and the distribution of site‐level dN/dS values was visualized to assess genome‐wide selection patterns.

### 2.4 Gene Family Expansion and Contraction Analysis

CAFE5 (De Bie et al. 2006) was employed to analyze gene family expansion and contraction using preprocessed data. The software utilizes a stochastic birth‐and‐death model to estimate the size of each gene family at each ancestral node and calculates family *p*‐values (based on a Monte Carlo resampling procedure) to determine whether significant expansion or contraction of gene families has occurred across different species. The input files included the gene family copy number results generated by OrthoFinder and the ultrametric tree file produced by r8s. Gene families with *p*‐values < 0.05 were considered to have undergone significant expansion or contraction. The results were visualized using the ggplot2 R package (Wickham and Chang 2016).

### 2.5 Identification of HGT‐Acquired Genes

The identification of HGT‐acquired genes followed the methodology described by Li et al. (2022). Briefly, protein sequences of the five Coccomorpha species were aligned against the RefSeq database (version released on May 5, 2023), protein sequences of 215 insect species, and the protein sequences of the Coccomorpha species themselves. Alignments were performed using DIAMOND BLASTP (Buchfink et al. 2015) with an e‐value threshold of 1e‐5 and max‐target‐seqs set to 1000. Subsequently, HGTfinder v1.0 (Shen et al. 2018) was used to calculate the Alien Index (AI) and out_pct (percentage of species in the OUTGROUP lineage) for each gene, both of which were defined and implemented in the research of Li et al. (Shen et al. 2018). Genes with out_pct ≥ 65 and AI > 0 were identified as candidate HGT‐acquired genes. To exclude potential contamination from non‐target organisms, contig sequences of each species were first aligned against the Genome Taxonomy Database (GTDB) using BLASTN to identify contigs potentially originating from bacteria, archaea, or other non‐target organisms (parameters: “‐outfmt 6 ‐evalue 1e‐5”). Next, contig sequences were aligned against the NCBI NT database (downloaded on April 24, 2023, from the NCBI FTP server) to extract taxonomic information, and contigs classified as non‐eukaryotic (e.g., bacteria, fungi, and viruses) were filtered out (parameters: “‐outfmt 6 ‐max_target_seqs 1 ‐evalue 1E‐5”). Additionally, Kraken2 (Wood et al. 2019), a k‐mer‐based tool, was employed to rapidly classify contigs and identify those assigned to microbial taxa with default parameters. Candidate genes located on contigs flagged as potential contaminants by any of the three methods were excluded. For further validation, candidate genes and their homologous sequences were aligned using MAFFT, and the alignments were trimmed using trimAl. Maximum likelihood trees were constructed using IQ‐TREE v2.2.0.3 (Nguyen et al. 2015), and the phylogenetic trees were visualized using the R package ggtree (Yu et al. 2017). Candidate genes nested within the phylogenetic trees of other species were considered likely acquired through HGT and were classified as HGT‐acquired genes.

### 2.6 Transcriptome Analysis

Transcriptomic SRA data for the five Coccomorpha species were retrieved from the NCBI database (Table S1). First, SRA files were converted to fastq format using the SRA Toolkit (Heldenbrand et al. 2017). Subsequently, fastp (Chen et al. 2018) was employed to perform quality control on the fastq data, removing adapter sequences and low‐quality reads to obtain high‐quality Clean data. The Clean data were then aligned to the respective reference genomes of the corresponding Coccomorpha species using Hisat2 (Kim et al. 2015). Finally, gene expression quantification was performed using featureCounts (Liao et al. 2014), and read counts were normalized to FPKM (Fragments Per Kilobase of transcript per Million mapped reads) values to standardize gene expression levels.

### 2.7 Identification of Detoxification‐Related Genes

To identify detoxification‐related gene families in Coccomorpha, Hidden Markov Model (HMM, v3.2.1) seed files were downloaded for detoxification gene families from Pfam (http://pfam.xfam.org/). These included the ABC domain (PF00005) of ABC transporters, the COE domain (PF00135) of the COE family, the Cytochrome P450 domain (PF00067) of the P450 gene family, the GST_C domain (PF00043) of the GST gene family, the UDPGT domain (PF00201) of the UGT gene family, the AOX domain (PF01315) of Aldehyde oxidase and xanthine dehydrogenase (AOX) gene family, the Glycosyl hydrolases family 18 domain (PF00704) of Chitinase Acidic (CHIA), the Epoxide hydrolase N terminus domain (PF06441) of Epoxide Hydrolase (EPHX), the PIF1‐like helicase domain (PF05970) of PIF1 5′ ‐to‐3′ DNA helicase (PIF), the Patched family domain (PF02460) of Patched Domain Containing (PTCHD), and the catalytic domain (PF00782) of protein tyrosine phosphatase (PTP). Protein sequences of the five Coccomorpha species were searched using HMMER v3.2.1 (Finn et al. 2011) with an E‐value threshold of 1e‐10. Proteins containing these domains were identified as members of the corresponding gene families.

For the construction of the COE gene family phylogenetic tree, COE protein sequences of *D. melanogaster* were retrieved from NCBI. First, multiple sequence alignment of COE protein sequences from the five Coccomorpha species and *D. melanogaster* was performed using MAFFT. The aligned sequences were then trimmed using trimAl, and a phylogenetic tree was constructed using IQ‐TREE. Finally, the tree was visualized using the R package ggtree.

### 2.8 Gene Functional Annotation and Enrichment Analysis

First, the CDS sequences of the five Coccomorpha species were aligned against the fly protein sequences from the KOBAS database (Bu et al. 2021) using DIAMOND blastx with the parameters “‐k 5 ‐e 0.00001 ‐p 10”. The blastx results were then annotated using KOBAS with the parameter “‐n 50.” Additionally, GO annotation of protein sequences for each species was performed using eggNOG‐mapper (Cantalapiedra et al. 2021) with the parameters “‐d euk ‐m diamond –resume.” For Pfam annotation, Pfam‐A.hmm was downloaded from InterProScan (https://www.ebi.ac.uk/interpro/) and used to search the protein sequences of each species via HMMER. Finally, enrichment analysis of target genes was conducted based on the annotation results using the R package clusterProfiler (Wu et al. 2021).

## 3 Results

### 3.1 Phylogenetic and Evolutionary Insights Into Coccomorpha

**FIGURE 1:**
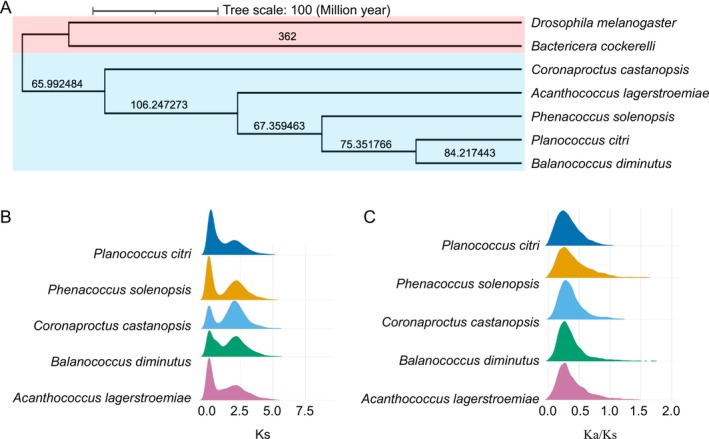
Evolutionary analysis of Coccomorpha. (A) Phylogenetic tree of Coccomorpha. Numbers on the branches represent divergence times. (B) Distribution of Ks values for orthologous genes in Coccomorpha. (C) Distribution of Ka/Ks ratios for orthologous genes in Coccomorpha.

To conduct a comparative genomic analysis of Coccomorpha species, chromosome‐level genomes of five Coccomorpha species were retrieved from NCBI, including *A. lagerstroemiae*, *B. diminutus* , *C. castanopsis*, *P. solenopsis*, and *P. citri* . To estimate the divergence times of these species, *D. melanogaster* and *B. cockerelli* were used as outgroups. Single‐copy orthologous genes were identified using OrthoFinder, and a phylogenetic tree was constructed based on these genes (Figure 1A). *C. castanopsis* was the first to diverge from the Coccomorpha lineage (approximately 333.18 mya), followed by *A. lagerstroemiae* (around 226.93 mya). *B. diminutus* and *P. citri* were the last to diverge within Coccomorpha (84.22 million years ago).

The ratios of Ka to Ks substitutions can be used to assess selective pressures acting on genes during evolution, thereby revealing patterns of adaptive evolution. Ka and Ks values were calculated for orthologous genes. The results indicated that the five Coccomorpha species underwent two WGD events (Figure 1B). The earliest WGD in five Coccomorpha species occurred between 88 and 95 mya, during the Late Cretaceous (Santonian to Cenomanian stages). This period was characterized by a greenhouse climate, with elevated atmospheric CO_2_ levels and global temperatures approximately 4°C–6°C higher than present. Concurrently, angiosperms underwent rapid evolutionary radiation, providing diverse ecological niches for herbivorous insects such as Coccomorpha. This WGD event may have been associated with environmental pressures (e.g., high temperatures, diversification of plant defensive compounds) and host‐adaptive evolution, laying the genomic foundation for subsequent species diversification and specialized feeding strategies in scale insects. Notably, this period also marked a key phase of adaptive radiation in many insect lineages (e.g., Coleoptera, Hymenoptera), suggesting that the WGD in scale insects may represent an evolutionary response to the ecological upheavals of the mid‐Cretaceous.

For the distribution of Ks values across different species, the Ks value distributions of *A. lagerstroemiae* and *P. solenopsis* were relatively broad, ranging from 0.00130763 to 8.99064 and from 0.000885161 to 8.40801, respectively, suggesting that these species accumulated more mutations during their evolutionary history. *A. lagerstroemiae* exhibited the widest Ks value range, reaching 8.99064, indicating a longer period of mutation accumulation in its genome. These distribution patterns reflect differences in evolutionary history and adaptive selective pressures among the species. The Ka/Ks distributions of orthologous genes in Coccomorpha revealed that most genes across the five species had Ka/Ks values below 0.5, indicating that these genes were primarily under purifying selection (Figure 1C). Consistently, selection pressure analysis based on HyPhy further demonstrated that the majority of codon sites in single‐copy orthologous genes were under purifying selection across Coccomorpha species, providing additional evidence for the pervasive evolutionary constraint acting on these genes (Figure S1).

### 3.2 Gene Family Expansion and Contraction in Coccomorpha

**FIGURE 2:**
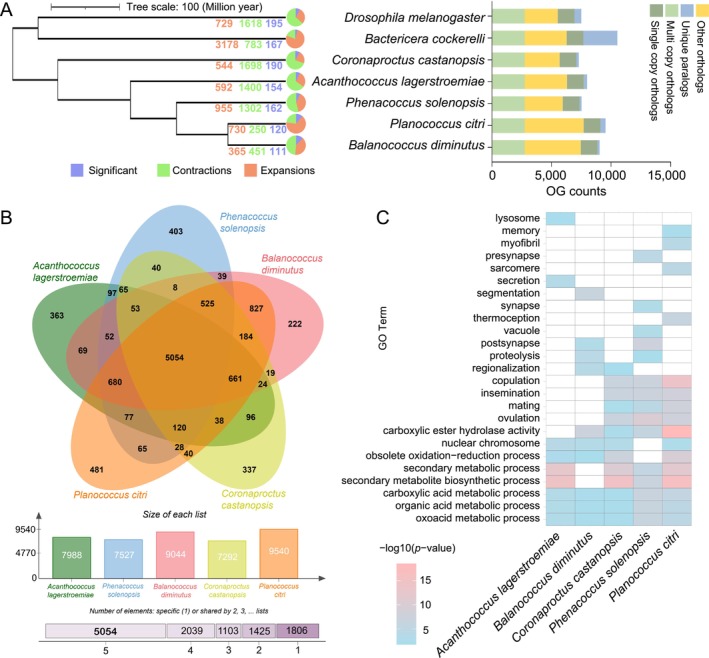
Gene family expansion and contraction in Coccomorpha. (A) The number of gene family expansions and contractions in the five Coccomorpha species. The pie chart represents the number of expanded, contracted, and significantly changed gene families. The bar chart shows the number of different types of orthologous groups (OGs) in the five Coccomorpha species. The red, green, and blue numbers on the branches of the phylogenetic tree represent the numbers of expanded, contracted, and significantly changed gene families in each species, respectively. (B) Shared gene families among the five Coccomorpha species. The central bar chart represents the number of OGs in each species, while the stacked bar chart below indicates the number of OGs present in 1–5 species. (C) GO enrichment analysis of significantly expanded gene families.

To analyze the unique and shared characteristics of gene families among Coccomorpha species, gene families were identified in the five Coccomorpha species and two outgroups. The gene families in the five Coccomorpha species exhibited varying degrees of expansion and contraction (Figure 2A). Among them, *P. solenopsis* showed the highest number of gene family expansions, with a total of 955 expanded gene families, suggesting that it may have undergone significant gene amplification during evolution to adapt to its specific ecological environment or physiological requirements. In contrast, *C. castanopsis* exhibited the highest number of gene family contractions, with a total of 1698 contracted gene families, along with the highest number of significantly changed gene families (190), indicating that its genome may have experienced large‐scale gene loss or functional reconfiguration during adaptive evolution. A total of 5054 gene families were shared among the five Coccomorpha species, which may be responsible for fundamental biological functions (Figure 2B). The number of gene families unique to a single species was second only to those shared by all five species (5054) and those shared by four species (2039), indicating substantial divergence among the five Coccomorpha species during evolution, potentially linked to their specific ecological adaptations, host preferences, or lifestyles.

Among the expanded gene families, GO terms such as oxoacid metabolic process, organic acid metabolic process, and carboxylic acid metabolic process were enriched to varying degrees in all five species. Other gene families showed species‐specific enrichment patterns. For example, secondary metabolite biosynthetic process was enriched in all species except *B. diminutus* , while lysosome was enriched only in *A. lagerstroemiae*, memory only in *P. citri* , and presynapse only in *P. solenopsis* (Figure 2C). Additionally, the contracted gene families in the five species exhibited distinct GO term enrichment patterns, suggesting that these species may have been subjected to different natural selection pressures during evolution (Table S2).

### 3.3 Identification of HGT‐Acquired Genes in Coccomorpha

**FIGURE 3:**
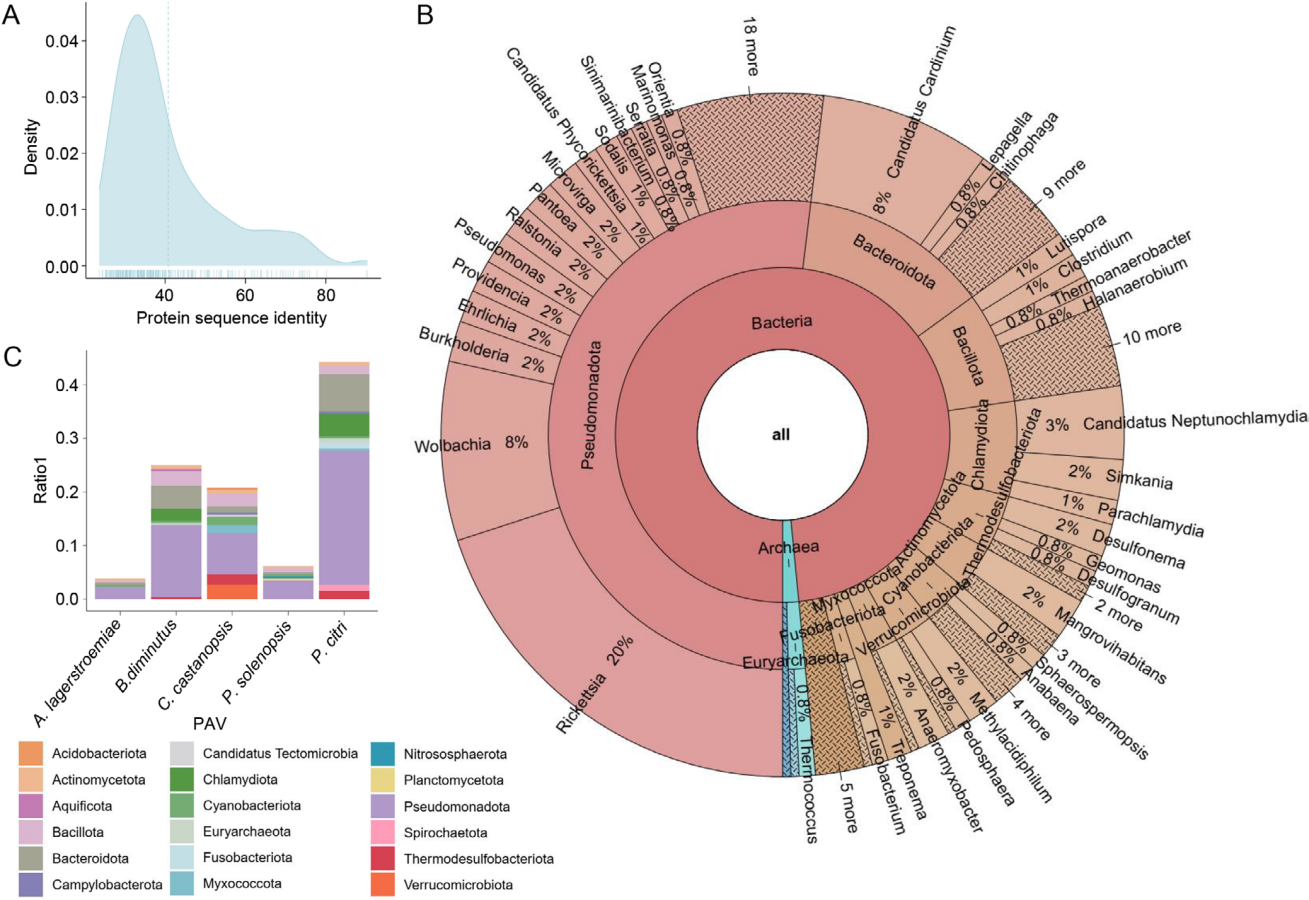
HGT‐acquired Genes in Five Coccomorpha Species. (A) Distribution of protein sequence similarity between HGT‐acquired genes in insect recipient genomes and their closest non‐metazoan donor sequences. (B) Putative donor distribution of HGT‐acquired genes. (C) Number of HGT‐acquired genes from different donors in the five Coccomorpha species.

HGT is a significant mechanism of genomic evolution, enabling the transfer of genes across species boundaries. By systematically identifying and analyzing HGT‐acquired genes in Coccomorpha insects, the molecular basis of their parasitic adaptation can be uncovered, providing new insights into the evolution and functional diversity of insect genomes. In this study, HGT‐acquired genes in five Coccomorpha species were identified. The numbers of HGT‐acquired genes in these species were as follows: 82 genes in *A. lagerstroemiae*, 65 genes in *B. diminutus* , 320 genes in *C. castanopsis*, 24 genes in *P. solenopsis*, and 115 genes in *P. citri* . These numbers far exceed those reported by Li et al. (Li et al. 2022) in Hemiptera, where the maximum number of HGT‐acquired genes in a single species was 152, with an average of 13 per species. The assembly quality of these genomes was assessed (Table S3). Although all genomes reached chromosome‐level quality, three out of the five had Complete BUSCOs below 95%. The number of HGT‐acquired genes located on contigs: 72 in *A. lagerstroemiae*, 266 in *C. castanopsis*, and 8 in *P. solenopsis* were also counted. No HGT‐acquired genes were found on contigs in *P. citri* or *B. diminutus* . These HGT‐acquired genes were attributed to potential contamination during genome assembly. After removing non‐eukaryotic sequences, including those from fungi, viruses, and microbes, a total of 260 HGT‐acquired genes were identified across the five species (*A. lagerstroemiae*: 10, *B. diminutus* : 65, *C. castanopsis*: 54, *P. solenopsis*: 15, *P. citri* : 115) (Table S4). None of these genes were located on contigs. The protein sequence similarity was further examined between the HGT‐acquired genes in insect recipients and their closest non‐metazoan homologs. The similarity values ranged from 23.6% to 90%, with an average of 40.76% (Figure 3A), consistent with findings from Li et al. (2022). These results confirm the reliability of the 260 HGT‐acquired genes identified in this study.

Regarding the origins of HGT‐acquired genes, the majority were derived from Bacteria, while Archaea accounted for a smaller proportion (2%, 4 HGT‐acquired genes). Within Bacteria, Pseudomonadota (including genera such as *Wolbachia* and *Rickettsia*) contributed the highest proportion (53% of the bacterial‐derived genes, 135 HGT‐acquired genes), followed by Bacteroidota (13% of the bacterial‐derived genes, 34 HGT‐acquired genes) and Bacillota (8% of the bacterial‐derived genes, 20 HGT‐acquired genes). Notably, *Wolbachia* and *Rickettsia* contributed 8% (22 HGT‐acquired genes) and 20% (52 HGT‐acquired genes) of all the HGT‐acquired genes, respectively, highlighting their significant roles in HGT. Within Archaea, Euryarchaeota contributed 75% (3 HGT‐acquired genes) of the archaeal‐derived genes, while other groups such as *Conexivisphaera* contributed 25% (1 HGT‐acquired gene) of the archaeal‐derived genes (Figure 3B). In terms of the number of HGT‐acquired genes per species, *P. citri* had the most genes acquired from diverse groups, particularly from Pseudomonadota and Bacteroidota. *C. castanopsis* and *B. diminutus* had similar numbers of HGT‐acquired genes, with Pseudomonadota being the primary source. *A. lagerstroemiae* had the smallest HGT‐acquired genes, indicating limited acquisition of foreign genes, with a relatively scattered gene origin. *P. solenopsis* exhibited a more balanced gene origin, but its total number of HGT‐acquired genes was lower than that of other species (Figure 3C). These results suggest significant differences in the scale and sources of HGT among species, likely reflecting their ecological adaptation strategies during evolution.

### 3.4 Functional Analysis of HGT‐Acquired Genes in Coccomorpha

**FIGURE 4:**
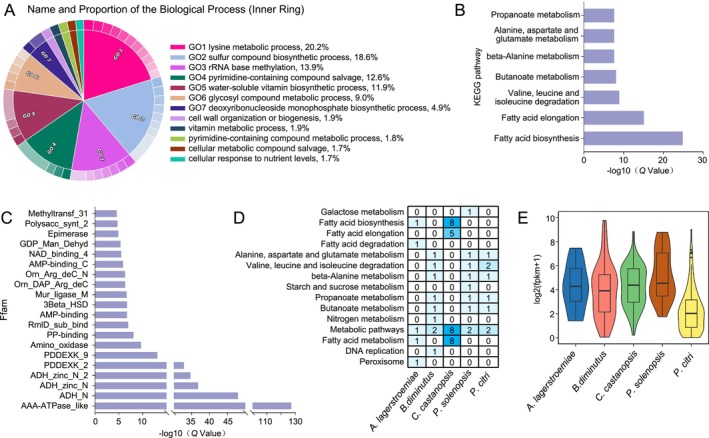
Functional analysis of HGT‐acquired genes in five Coccomorpha species. (A) GO enrichment analysis of HGT‐acquired genes in the Biological Process category. (B) KEGG pathway enrichment analysis of HGT‐acquired genes. (C) Pfam enrichment analysis of HGT‐acquired genes. (D) Number of HGT‐acquired genes across different pathways in the five Coccomorpha species. (E) Expression levels of HGT‐acquired genes in different species.

To further understand the functions of HGT‐acquired genes identified in the five Coccomorpha species, enrichment analysis was performed using all HGT‐acquired genes from these species, with all genes from the species serving as the background. GO enrichment analysis revealed that these genes were primarily enriched in biological processes such as lysine metabolic process, glycosaminoglycan biosynthetic process, and water‐soluble vitamin biosynthetic process. These enriched processes suggest that HGT may play a significant role in amino acid metabolism, carbohydrate compound synthesis, and vitamin metabolism (Figure 4A). Additionally, enrichment in dendritic structure, mitochondrial intermembrane space, and external encapsulating structure indicates a close association between HGT and cellular structure and organelle function (Figure S2). At the molecular function level, RNA methyltransferase activity and DNA/RNA binding activity were also significantly enriched, suggesting that these genes may be involved in key molecular mechanisms such as post‐transcriptional modification and gene expression regulation (Figure S3). KEGG pathway enrichment highlighted metabolic pathways including pyruvate metabolism, amino acid metabolism (e.g., alanine, aspartate, and glutamate metabolism), and fatty acid elongation, indicating that HGT‐acquired genes may be closely related to cellular energy production and amino acid synthesis. Pfam enrichment results primarily involved molecular functions such as transferase activity, NAD binding, and ATP binding, suggesting that these genes may play roles in post‐transcriptional modification and cellular signaling processes.

Furthermore, the number of HGT‐acquired genes across pathways in the five Coccomorpha species was visualized (Figure 4D). In *C. castanopsis*, HGT‐acquired genes were predominantly concentrated in pathways such as fatty acid biosynthesis, fatty acid elongation, metabolic pathways, and fatty acid metabolism, with most being associated with lipid metabolism. This enrichment suggests that *C. castanopsis* may enhance its fatty acid synthesis and elongation capabilities through HGT, thereby supporting energy storage and cell membrane biosynthesis. To investigate the expression of these HGT‐acquired genes across species, all available transcriptomic data were downloaded from public databases and their expression levels quantified. HGT‐acquired genes exhibited varying degrees of expression across species. Notably, the expression distribution of these genes was relatively uniform in *B. diminutus* , while *P. solenopsis* showed relatively higher expression levels of HGT‐acquired genes (Figure 4E). These results indicate that HGT‐acquired genes may perform different functions in different species.

### 3.5 Detoxification‐Related Genes in Coccomorpha

**FIGURE 5:**
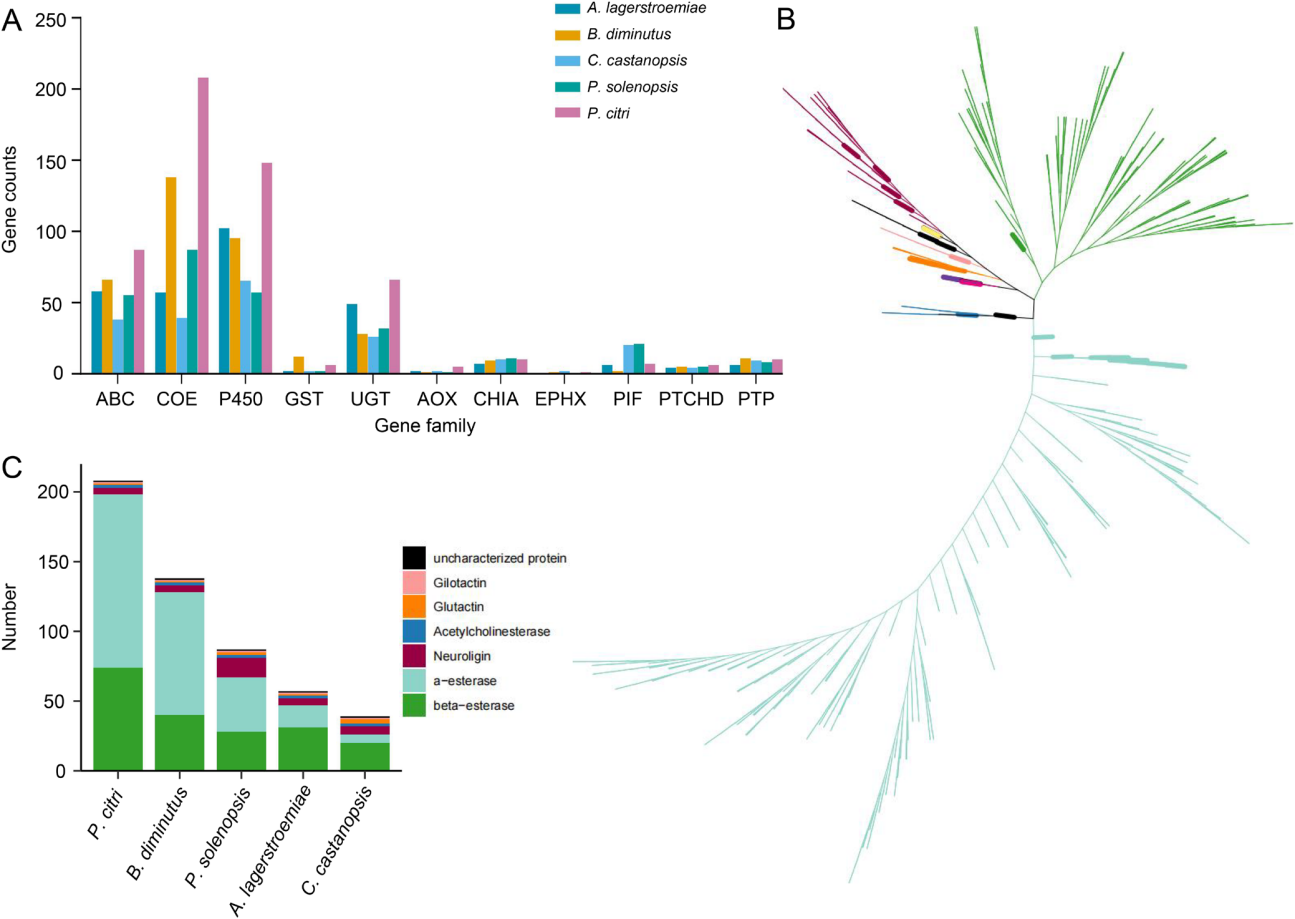
Detoxification‐related genes in five Coccomorpha species. (A) Number of genes in detoxification‐related gene families across the five Coccomorpha species. (B) Phylogenetic tree of the COE gene family in the five Coccomorpha species. (C) Number of genes in different subfamilies of the COE gene family across the five Coccomorpha species.

As significant pests, detoxification‐related genes in Coccomorpha play a crucial role in adapting to pesticide pressure and environmental changes. Identifying these genes is essential for understanding the mechanisms of pesticide resistance in Coccomorpha. In this study, detoxification‐related genes were identified in five Coccomorpha species (Figure 5A and Table S5). Among them, *ABC*, *COE*, *P450*, and *UGT* are the four largest detoxification‐related gene families, with each family containing over 200 members across the five species, indicating their potential importance in detoxification processes. In contrast, the *EPHX* gene family is relatively small, with only four members in total (two in *C. castanopsis*, and one each in *P. citri* and *B. diminutus* ). This suggests that the *EPHX* gene family may play a limited role in detoxification in these species or that these species are less sensitive to certain environmental toxins. Among the five Coccomorpha species, *C. castanopsis* has the fewest detoxification‐related genes (217, compared to an average of 342 across the five species), while *P. citri* has the most (554). This difference may reflect variations in detoxification capabilities among species. The *COE* gene family exhibits the most significant variation in gene number across the five species, with the fewest in *C. castanopsis* (39) and the most in *P. citri* (208).

This study constructed a phylogenetic tree for the COE gene family (Figure 5B and Figure S4). It was reported that organophosphate esters (OPEs) as a major component of organophosphate pesticides, inhibit acetylcholinesterase (AChE) at cholinergic synapses in both the central and peripheral nervous systems, leading to disruption of neurotransmission and eventual death (Marrs 1993). α‐esterases can bind and slowly hydrolyze OPEs (Birner‐Gruenberger et al. 2012), thereby conferring significant protective effects on insects (Jackson et al. 2013). However, the α‐esterase subfamily is the largest; this may explain the differences in pesticide resistance among the five species. Interestingly, *C. castanopsis* has a notably small proportion of α‐esterase genes (6, accounting for 15% of its COE genes) (Figure 5C and Table S6), which may be related to its limited exposure to pesticide selection pressure.

## 4 Discussion

Coccomorpha, as significant pests, have garnered considerable attention due to their remarkable environmental adaptability. In recent years, the advancement of high‐throughput sequencing technologies has led to a gradual increase in genomic data for Coccomorpha species, providing valuable resources for comparative genomic analyses (Huang et al. 2024; Ross et al. 2024; Ross and Watson 2024; Li et al. 2020). In this study, five high‐quality, chromosome‐level genomes from public databases were collected. Based on the phylogenetic tree (Figure 1A), *C. castanopsis* is the most distantly related to the other four Coccomorpha species, followed by *A. lagerstroemiae*. Notably, *C. castanopsis* exhibited significant differences from the other species in multiple analyses. For instance, it had the highest number of contracted gene families among the five Coccomorpha species, suggesting potential gene loss or functional reconfiguration during its evolutionary history. In addition, it will be worth noting that although *D. melanogaster* (Diptera) and *B. cockerelli* (Hemiptera) appeared as a sister clade in the phylogenetic tree, this topology likely resulted from the limited number of outgroup taxa and potential long‐branch attraction. These two species were used only as outgroups for rooting purposes, and their inferred relationship does not represent the true evolutionary history. To further explore the adaptive mechanisms of these species, the expanded gene families were analyzed. Among the expanded gene families, the most enriched GO terms included oxoacid metabolic process, organic acid metabolic process, and carboxylic acid metabolic process, which were enriched in all five species. These GO terms have been shown to be associated with the folate cycle and the kynurenine pathway. To protect themselves from oxidative stress caused by pesticides and other toxic compounds, insects have evolved mechanisms involving the synergistic action of antioxidant enzymes. Folate, as an antioxidant compound, has demonstrated significant potential in scavenging reactive oxygen species (ROS) and has been proven to mitigate oxidative stress induced by the pesticide hexaconazole in silkworms (Ashraf et al. 2022). This suggests that scale insects may have evolved expanded antioxidant mechanisms, such as the antioxidant effects of folate, to counteract oxidative stress caused by pesticides and other foreign compounds.

HGT is a significant evolutionary force shaping the genomes of both prokaryotes and eukaryotes. A growing body of research indicates that HGT‐acquired genes are associated with insect adaptability. For example, studies have shown that male diamondback moths lacking the HGT‐acquired gene *LOC105383139* exhibit significantly reduced pursuit of females (Li et al. 2022). In silkworms, the HGT‐acquired gene *BGIBMGA007146* is involved in the degradation, modification, and binding of toxins (Meng et al. 2009), *BGIBMGA011204* enhances metabolic pathways to evade toxic targets (Faulkner 1955), and *BGIBMGA005696* participates in replacing target enzymes with resistance enzymes (Daimon et al. 2008). In this study, HGT events in five Coccomorpha species were identified. After filtering HGT‐acquired genes, all genes located on contigs were removed. However, this also means that HGT genes located in fragmented regions of the genome may have been missed. The relatively low BUSCO completeness in some genomes (Table S3) suggests potential genome assembly incompleteness, which could lead to an underestimation of HGT events. Therefore, our results likely represent a conservative estimate, and this study anticipates that higher‐quality genome assemblies will enable the identification of a more comprehensive set of HGT‐acquired genes. Previous studies have shown that Hemiptera exhibits a higher number of horizontally transferred genes (HGTs) than most other insect orders, second only to Lepidoptera, with up to 170 HGTs identified in some species (Li et al. 2022). In this study, 10–115 HGTs were identified in per species, a range consistent with previous reports, supporting the robustness of our species selection and HGT detection pipeline. Notably, species within Coccomorpha exhibited relatively high numbers of HGTs. This may be related to their highly specialized ecological niches—Coccids show strong host specificity, with about 64% of species recorded from only a single plant family (Lin et al. 2010)—combined with their phloem‐feeding lifestyle and sedentary behavior (Kapranas 2012). These factors likely promote long‐term and intimate associations with endosymbiotic bacteria and environmental microbes, creating favorable conditions for cross‐kingdom gene transfer. It is noteworthy that current information regarding the microbial environment of Coccomorpha remains limited. Metagenomic sequencing could be employed to further enhance the evolutionary interpretation of HGT events, including the interactions between Coccomorpha and their symbiotic bacteria, as well as niche specialization.

Furthermore, previous research using transcriptomic data identified 22 HGT‐acquired genes in *P. citri* (Husnik et al. 2013), whereas our study identified 115 HGT‐acquired genes in this species. However, most of these genes exhibited low expression levels (Figure 4E), indicating that the majority of HGT‐acquired genes in *P. citri* are not expressed and are therefore difficult to detect in transcriptome‐based HGT identification. Functionally, the HGT‐acquired genes were primarily enriched in pathways and GO terms related to nutrient metabolism. It has been reported that Coccomorpha, which feed exclusively on the phloem of host plants, rely on symbiotic bacteria to provide essential nutrients. The phloem is rich in sugars but deficient in amino acids and vitamins, and symbiotic bacteria in Coccomorpha play a crucial role in supplying these nutrients (García Morales et al. 2016; Rosenblueth et al. 2012). Moreover, Coccomorpha acquires genes from these symbiotic bacteria or other bacteria and expresses these HGT‐acquired genes to compensate for the loss of nutrient and structural synthesis pathways in their reduced symbiotic genomes (Husnik et al. 2013; Husnik and McCutcheon 2016; Szabó et al. 2017; Bublitz et al. 2019). This genetic complementation further highlights the critical role of HGT in enabling Coccomorpha to adapt to its unique ecological niches. Notably, although a large number of HGT‐acquired genes were identified in this study, some exhibited low expression levels, which may be related to functional redundancy or environmentally dependent expression regulation. However, the functional roles of these HGT genes remain largely hypothetical. Future studies integrating high‐quality genomic data, transcriptomic profiling, and functional validation techniques, such as RNA interference (RNAi) or CRISPR‐Cas9 genome editing, are essential to confirm their specific contributions to nutrient metabolism, detoxification, and overall adaptability in Coccomorpha. These approaches will help elucidate the evolutionary significance and biological relevance of HGT events in this group.

During their long evolutionary history, Coccomorpha pests have adapted to various environmental pressures, with detoxification‐related gene families playing a crucial role in the development of pesticide resistance. This study identified *ABC*, *COE*, *P450*, and *UGT* as the most prominent detoxification gene families in Coccomorpha, with each species harboring over 200 members in these families, suggesting their central role in detoxification metabolism. In contrast, the *EPHX* gene family was found in only a few species and in limited numbers, indicating that its function in Coccomorpha may be restricted or specific to certain toxins. Significant differences were observed in the number and composition of detoxification genes among species. For instance, *P. citri* possesses the highest number of detoxification‐related genes, while *C. castanopsis* has the fewest. These differences may be linked to variations in ecological niches, dietary preferences, or historical exposure to pesticides. Notably, the *COE* gene family exhibited the most pronounced variation across species, with *C. castanopsis* having significantly fewer COE genes than other species. This is particularly evident in the α‐esterase subfamily, which is closely associated with pesticide resistance, where *C. castanopsis* has a notably lower proportion of genes. Previous studies have shown that the expansion of α‐esterase is linked to pesticide resistance in various insects (Jackson et al. 2013), which may explain the differences in pesticide resistance among Coccomorpha species. In particular, *C. castanopsis*, a recently emerged oligophagous species, feeds primarily on plants from the Fagaceae family and exhibits a significantly reduced number of *COE* genes, including α‐esterases. This reduction may not merely reflect a response to lower pesticide exposure but rather an adaptive genomic streamlining driven by its narrow host range. Specialization on a limited set of host plants likely reduces the need for a broad‐spectrum detoxification system, allowing the species to rely on more specific and efficient mechanisms to counter host‐derived allelochemicals. However, such specialization may come at the cost of reduced flexibility in dealing with novel or variable chemical environments.

In contrast, generalist species like *P. citri* , which feed on host plants from 334 genera across 112 families (García Morales et al. 2016), possess a higher number of detoxification genes and may have a survival advantage under diverse host plant or pesticide pressures. While these patterns suggest possible adaptive responses to different ecological contexts, the expansion or contraction of detoxification gene families may also result from other evolutionary processes, such as genetic drift or lineage‐specific duplications. Therefore, adaptive evolution should be considered as one possible explanation rather than a definitive cause. Further functional and population‐level analyses would be needed to confirm adaptive selection acting on specific detoxification genes. Overall, the composition and evolutionary patterns of detoxification gene families vary significantly among Coccomorpha species, reflecting their adaptive strategies in different ecological environments. These findings provide important insights into the detoxification mechanisms and pesticide resistance evolution of Coccomorpha pests and offer potential molecular targets for developing pest control strategies.

## Author Contributions

**Xu Wang:** funding acquisition (lead), methodology (lead), resources (equal), validation (equal), visualization (supporting), writing – original draft (equal), writing – review and editing (lead). **Yi‐Xin Huang:** conceptualization (lead), data curation (supporting), formal analysis (lead), funding acquisition (equal), investigation (lead), methodology (equal), project administration (equal), visualization (lead), writing – original draft (lead), writing – review and editing (lead). **Ji‐Mu Lv:** investigation (equal), methodology (equal), writing – review and editing (equal). **Cheng‐Yang Ding:** formal analysis (equal), investigation (equal). **Xin‐Yi Zheng:** investigation (equal), visualization (equal). **Xing‐Jie Liu:** investigation (equal), writing – review and editing (equal). **Xiao‐Nan Chen:** investigation (equal), resources (equal). **Shun‐Hai Yu:** investigation (equal), resources (equal). **Yin‐Feng Meng:** formal analysis (equal), visualization (equal). **Hao‐Yuan Hu:** funding acquisition (equal), methodology (equal), project administration (equal), resources (equal), writing – review and editing (equal).

## Conflicts of Interest

The authors declare no conflicts of interest.

## Supporting information


**Figure S1:** Density distribution of codon‐level dN/dS values inferred by HyPhy.

**Figure S2:** The enrichment analysis for HGT‐acquired genes in the category of Cellular Component (CC).

**Figure S3:** The enrichment analysis for HGT‐acquired genes in the category of Molecular Function (MF).

**Figure S4:** The phylogenetic tree of COE gene family.

**Table S1:** The transcriptome information employed in this study.

**Table S2:** GO enrichment analysis of significantly contracted gene families.

**Table S3:** BUSCO assessment of the genomes of five Coccomorpha species.

**Table S4:** HGT‐acquired genes identified in this study.

**Table S5:** The detoxification‐related genes identified in this study.

**Table S6:** Detailed information on the COE gene family.

## Data Availability

The data and results supporting this study are available within the article and in the Data S1.
