# Supplementary figures and images for: Genomic Evolution and Patterns of Horizontal Gene Transfer in Coccomorpha Species
Source: Ecol Evol. 2025 Oct 9;15(10):e72158. doi: 10.1002/ece3.72158 (PMC12510726; doi:10.1002/ece3.72158)

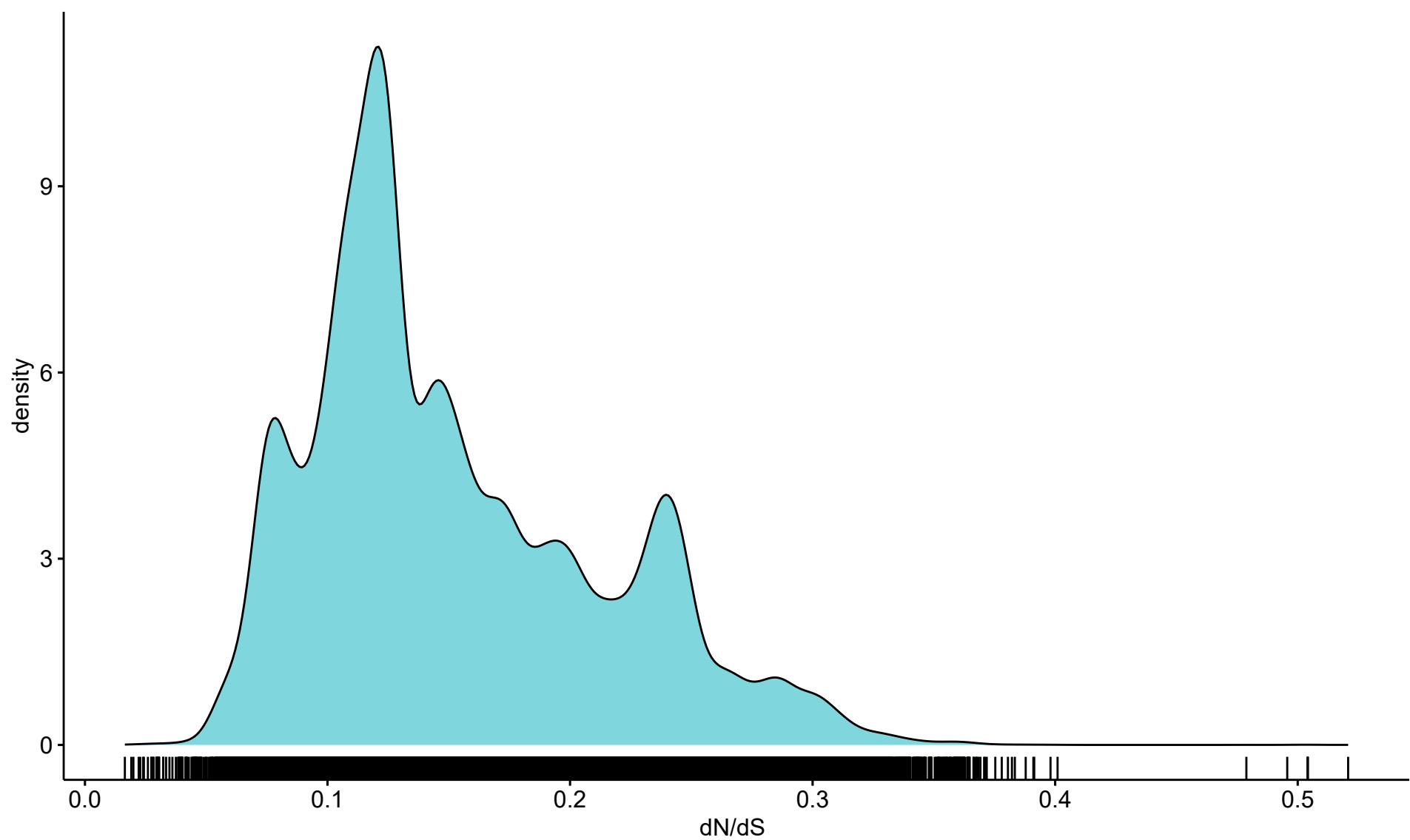

Supplement: Supplementary file 1 — Figure S1: Density distribution of codon‐level dN/dS values inferred by HyPhy. [file ECE3-15-e72158-s005.pdf]

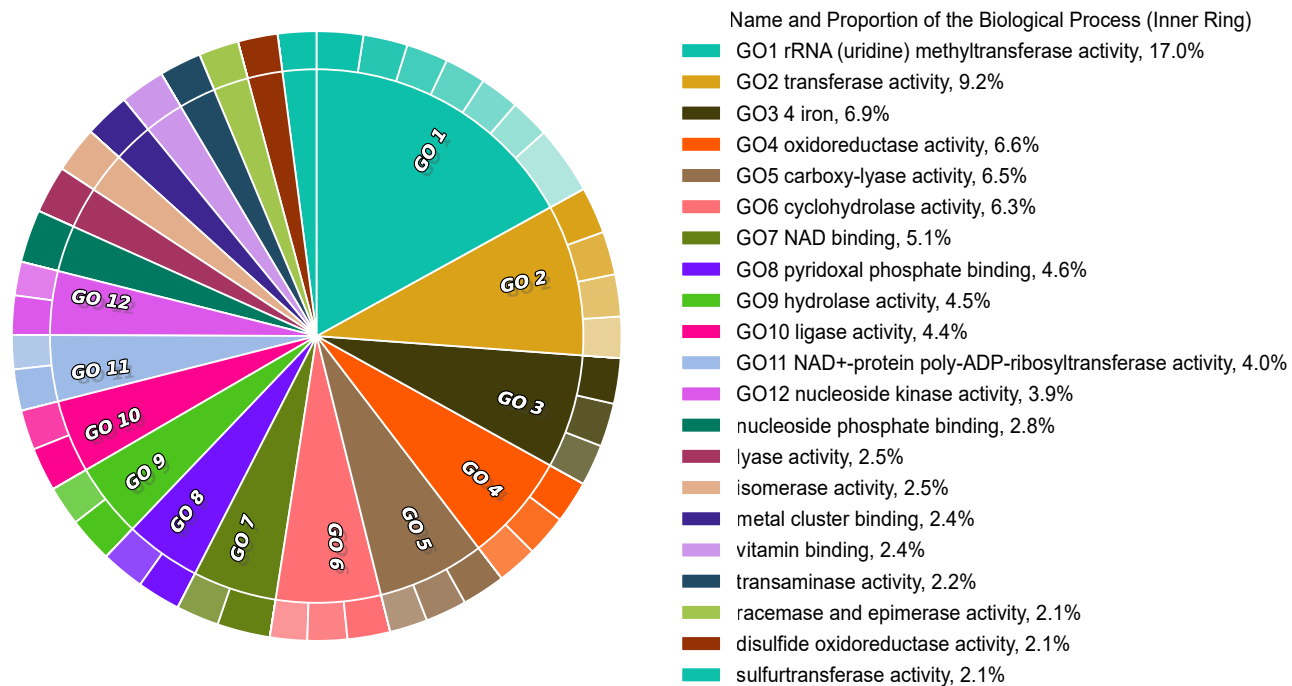

Supplement: Supplementary file 3 — Figure S3: The enrichment analysis for HGT‐acquired genes in the category of Molecular Function (MF). [file ECE3-15-e72158-s003.pdf]

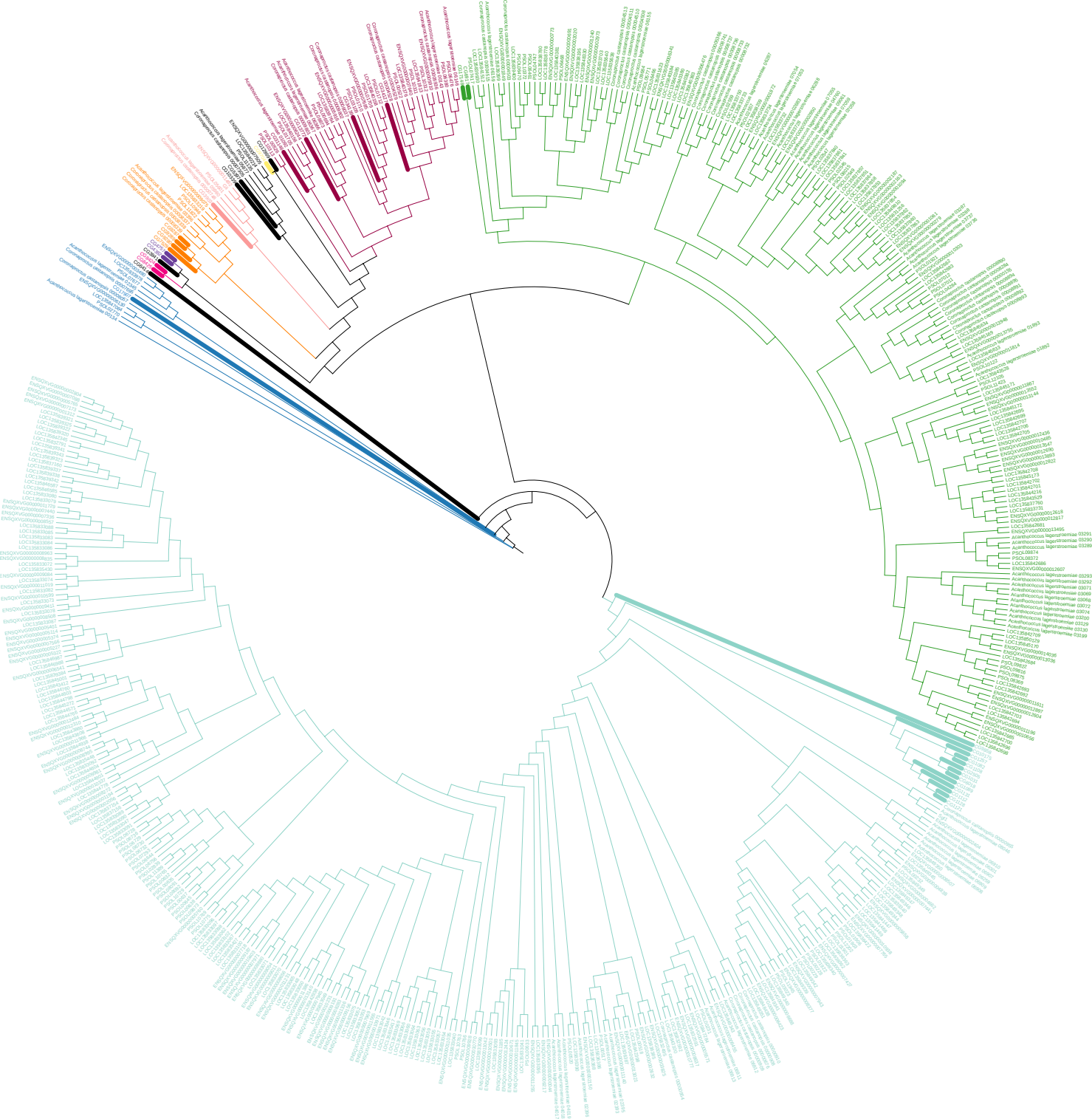

Supplement: Supplementary file 4 — Figure S4: The phylogenetic tree of COE gene family. [file ECE3-15-e72158-s002.pdf]
