# Supplementary material for: Genomic Evolution and Patterns of Horizontal Gene Transfer in Coccomorpha Species
Source: Ecol Evol. 2025 Oct 9;15(10):e72158. doi: 10.1002/ece3.72158 (PMC12510726; doi:10.1002/ece3.72158)
Supplement: Supplementary file 2 — Figure S2: The enrichment analysis for HGT‐acquired genes in the category of Cellular Component (CC). [file ECE3-15-e72158-s008.pdf]

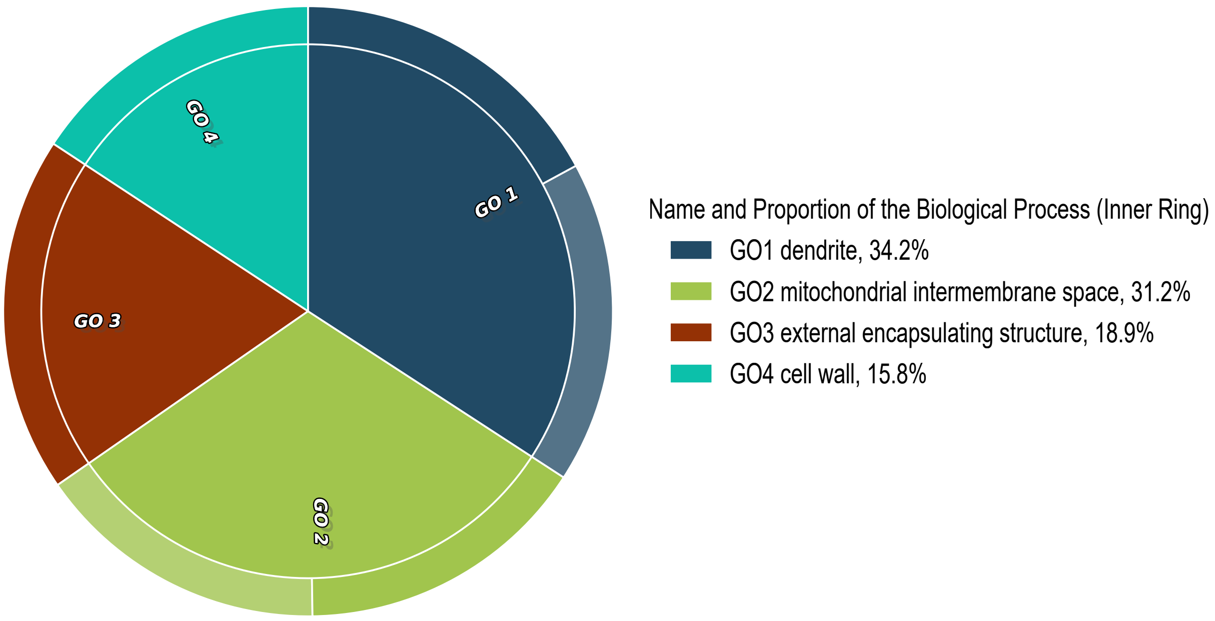
GO 1
GO 2
GO 3
GO 4
Name and Proportion of the Biological Process (Inner Ring)
GO1 dendrite, 34.2%
GO2 mitochondrial intermembrane space, 31.2%
GO3 external encapsulating structure, 18.9%
GO4 cell wall, 15.8%
